# Computer-controlled closed-loop drug infusion system for automated hemodynamic resuscitation in endotoxin-induced shock

**DOI:** 10.1186/s12871-017-0437-9

**Published:** 2017-10-23

**Authors:** Kazunori Uemura, Toru Kawada, Can Zheng, Meihua Li, Masaru Sugimachi

**Affiliations:** Department of Cardiovascular Dynamics, National Cerebral and Cardiovascular Center, 5-7-1 Fujishirodai, Suita, 565-8565 Japan

**Keywords:** Septic shock, Hemodynamics, Resuscitation, Computer, Closed-loop control

## Abstract

**Background:**

Hemodynamic resuscitation in septic shock requires aggressive fluid replacement and appropriate use of vasopressors to optimize arterial pressure (AP) and cardiac output (CO). Because responses to these drugs vary between patients and within patient over time, strict monitoring of patient condition and repetitive adjustment of drug dose are required. This task is time and labor consuming, and is associated with poor adherence to resuscitation guidelines. To overcome this issue, we developed a computer-controlled closed-loop drug infusion system for automated hemodynamic resuscitation in septic shock, and evaluated the performance of the system in a canine model of endotoxin shock.

**Methods:**

Our system monitors AP, CO and central venous pressure, and computes arterial resistance (R), stressed blood volume (V) and Frank-Starling slope of left ventricle (S). The system controls R with noradrenaline (NA), and V with Ringer’s acetate solution (RiA), thereby controlling AP and CO. In 4 dogs, AP and CO were measured invasively. In another 4 dogs, AP and CO were measured less invasively using clinically acceptable modalities, aiming to make the system clinically feasible. In all 8 dogs, endotoxin shock was induced by injecting *Escherichia coli* lipopolysaccharide, which significantly decreased AP from 95 (91–108) to 43 (39–45) mmHg, and CO from 112 (104–142) to 62 (51–73) ml·min^−1^·kg^−1^. The system was then connected to the dogs, and activated. System performance was observed over a period of 4 h.

**Results:**

Our system immediately started infusions of NA and RiA. Within 40 min, RiA increased V to target level, and NA maintained R at target level, while S was concomitantly increased. These resulted in restoration of AP to 70 (69–71) mmHg and CO to 130 (125–138) ml·min^−1^·kg^−1^. Median of absolute performance error, an index of precision of control, was small in AP [2.5 (2.1–4.5) %] and CO [2.4 (1.4–5.5) %], which were not increased even when the variables were measured less invasively.

**Conclusions:**

In a canine model of endotoxin shock, our system automatically improved and maintained AP and CO at their target values with small performance error. Our system is potentially an attractive clinical tool for rescuing patients with septic shock.

**Electronic supplementary material:**

The online version of this article (10.1186/s12871-017-0437-9) contains supplementary material, which is available to authorized users.

## Background

Sepsis is a major healthcare problem worldwide, and may lead to septic shock [1]. The last two decades have seen substantial improvements in outcomes of this serious condition. Septic shock, however, remains the most common cause of death among critically ill patients in intensive care units, with mortalities ranging from 10 to 30% [2, 3]. Several clinical studies indicate that implementation of the Surviving Sepsis Campaign (SSC) guidelines is associated with reduction in mortality in this condition [4]. However, adherence to the guideline-based hemodynamic resuscitation and infection control is still poor [5].

The SSC guidelines recommend aggressive fluid replacements and appropriate use of vasopressors to optimize systemic arterial pressure (AP), blood flow, [or cardiac output (CO)], and oxygen delivery in septic shock [1]. Because responses to these drugs vary between patients and within patient over time, strict monitoring of patient condition and repetitive adjustment of drug doses are usually required. These tasks are time and labor consuming, and are associated with poor adherence to SSC guidelines [6]. One potential way to ease these tasks is to automate hemodynamic resuscitation utilizing closed-loop control schemes [7]. However, there is little attempt to implement this scheme in patients with septic shock. Only one clinical study conducted closed-loop control of vasopressor dose in septic shock patients [8]. In that study, closed-loop automated control of the weaning from vasopressor support (noradrenaline, NA) significantly reduced the duration of shock and the total dose of NA required, strongly suggesting the potential benefit of using closed-loop control schemes in resuscitating septic hemodynamics. However, no closed-loop system has been developed to automate the entire process of hemodynamic resuscitation in subjects with septic shock.

We have developed a closed-loop automated cardiovascular drug infusion system for simultaneous control of AP, CO, and left heart filling pressure (pulmonary wedge pressure, PWP) in acute heart failure [9–11]. From AP, CO, and PWP, our system estimates systemic arterial resistance (R), total stressed blood volume (V) and Frank-Starling slope of the left ventricle (S) based on a framework of circulatory equilibrium [12, 13]. Since acute heart failure is characterized with abnormally increased R and V, and with depressed S, our system was previously designed to control R with a vasodilator, V with diuretics (or fluids), and S with an inotrope, thereby controlling AP, CO, and PWP [9–11]. In canine models of acute heart failure, our system has successfully controlled AP, CO, and PWP with good accuracy and stability [9–11], which suggests the potential clinical utility of the system. Furthermore, the framework of circulatory equilibrium is derived from Guyton’s original framework [12–14], which is applicable to the analysis of septic hemodynamics [15, 16]. Taken all together, we hypothesized that our closed-loop drug infusion system can be expanded and developed to resuscitate hemodynamics in subjects with septic shock.

The aim of this study was to develop a computer-controlled closed-loop drug infusion system for automated hemodynamic resuscitation in septic shock, and evaluate the performance of the newly developed system in canine models of endotoxin shock.

## Methods

### Design of automated drug infusion system for hemodynamic resuscitation in septic shock

**Fig. 1:**
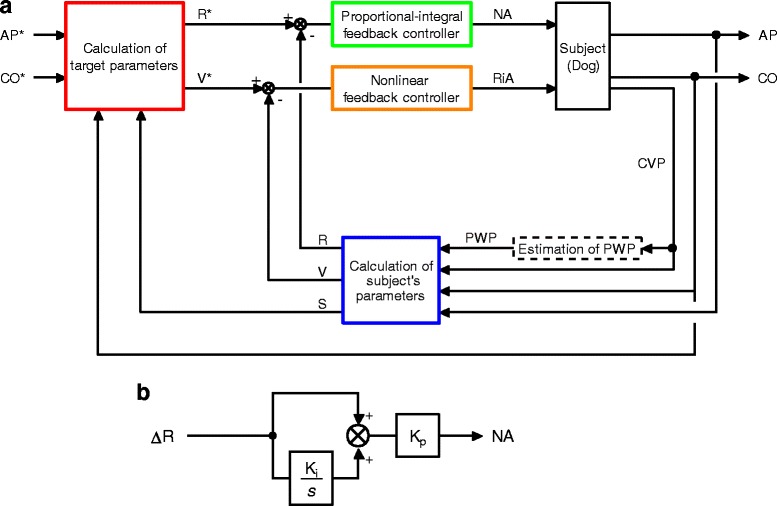
Closed-loop drug infusion system for automated hemodynamic resuscitation in septic shock. **a** Block diagram of the system for simultaneous control of mean arterial pressure (AP) and cardiac output (CO) in septic shock. AP* and CO* represent target AP and target CO, respectively. From these target variables, target values of systemic arterial resistance (R*) and stressed blood volume (V*) are determined (red rectangle). Subject’s R, V, and Frank-Starling slope of the left ventricle (S) are calculated from AP, CO, central venous pressure (CVP) and pulmonary wedge pressure (PWP) (blue rectangle). The proportional-integral controller adjusts infusion rate of noradrenaline (NA) to minimize the difference between R and R* (green rectangle). A non-linear controller adjusts infusion of Ringer’s acetate (RiA) to minimize the difference between V and V* (orange rectangle). **b** Block diagram of the proportional-integral controller. Ki and Kp represent the integral and proportional gain constants, respectively. s is a Laplace operator

Figure 1 is a schematic illustration of the system developed in this study. The system controls the infusion of NA and fluid (Ringer’s acetate, RiA) to optimize AP and CO using a negative feedback mechanism. PWP is estimated from central venous pressure (CVP) [17] (Fig. 1a) (details are provided in Appendix 1, Additional file 1). The subject’s R, V and S are continuously calculated from AP, CO, CVP and PWP (blue rectangle in Fig. 1a) (see Appendix 1, Additional file 1). User of the system defines target values for AP (AP*) and for CO (CO*), and inputs them into the system. Based on AP*, CO*, and the subject’s CO and S, the system calculates target values for R (R*), and for V (V*) (red rectangle in Fig. 1a) (see Appendix 2, Additional file 2). Calculations of R* and V* are repeated every minute. The subject’s R and V are compared with R* and V*, respectively. To minimize the difference between R* and R (ΔR = R*-R), a proportional-integral feedback controller adjusts the infusion rate of NA (green rectangle in Fig. 1a). In the proportional-integral feedback controller (Fig. 1b), ΔR and the difference integrated with an integral gain (Ki) are summed and scaled by a proportional gain (Kp) to give the infusion rate of NA. We determined the gain constants for NA infusion [Ki = 0.83 s^−1^, Kp = 0.42 μg·kg^−1^·min^−1^ (mmHg·min·kg·ml^−1^)^−1^] based on open-loop response of R to NA infusion in our preliminary experiments (see Appendix 3, Additional file 3). To minimize the difference between V* and V (ΔV = V*-V), a nonlinear feedback controller (orange rectangle in Fig. 1a) adjusts the infusion of RiA based on the following “if-then” rules:

$$ \mathrm{If}\ \mathrm{CO}\le {\mathrm{CO}}^{\ast }\ \mathrm{and}\ \Delta \mathrm{V}\ge 0\ \mathrm{ml}\cdotp {\mathrm{kg}}^{-1},\mathrm{then}\  \mathrm{infuse}\ \mathrm{RiA}\ \mathrm{at}\ 15\ \mathrm{ml}\cdotp {\mathrm{min}}^{-1}. $$

$$ \mathrm{If}\ \mathrm{CO}>{\mathrm{CO}}^{\ast }\ \mathrm{and}\ \Delta \mathrm{V}\ge 1\ \mathrm{ml}\cdotp {\mathrm{kg}}^{-1},\mathrm{then}\  \mathrm{infuse}\ \mathrm{RiA}\ \mathrm{at}\ 15\ \mathrm{ml}\cdotp {\mathrm{min}}^{-1}. $$

These adjustment processes operate continuously in parallel until the differences disappear.

### Animals

We used 8 mongrel dogs for laboratory use (3 males and 5 females; Kitayama Labes, Gifu, Japan). The mean body weight was 25 ± 3 kg, and the mean age was 4 ± 3 years. The experiments conformed to the Guide for the Care and Use of Laboratory Animals published by the US National Institutes of Health (NIH Publication No. 85–23, revised 1996). All protocols were approved by the Animal Subjects Committee of the National Cerebral and Cardiovascular Center.

In 4 dogs (group A), AP and CO were measured invasively. In the other 4 dogs (group B), AP and CO were measured less invasively to test the clinical feasibility of the system [11].

### Preparation

In group A, after anesthesia was induced with thiamylal sodium (25 mg·kg^−1^), the animal was intubated endotracheally and ventilated artificially. An appropriate level of anesthesia was maintained by continuous inhalation of 2.0% isoflurane. Using aseptic technique, a left thoracotomy and a pericardial incision were performed. An aortic flow probe (24PAX, Transonics Systems, Ithaca, NY) was placed on the ascending aorta as described previously [18]. Probe cable was exteriorized through the animal’s back. The chest wall was closed, and the animal was allowed to recover for 4 weeks after the surgery. Animals in group B did not undergo surgical placement of the aortic probe.

In both groups, the animals were anesthetized and mechanically ventilated in left lateral recumbent position. Surface ECG was recorded. A 10-F sheath introducer was placed in the right jugular vein. The side-port of the introducer was connected to a pressure transducer (DX-200, Nihon Kohden, Tokyo, Japan) to measure CVP. An infusion pump (CFV-3200, Nihon Kohden, Tokyo, Japan) for administering NA (Daiichi-Sankyo, Tokyo, Japan) and a roller pump (Minipulse 3, Gilson, Middleton, WI) for administering RiA (Veen-F, Kowa Pharmaceutical Co Ltd., Tokyo, Japan) were attached to a catheter (8F) placed in the right femoral vein. These pumps were controlled with a laboratory computer (Computer 1) (MA20V, NEC, Tokyo, Japan). A catheter (8F) was placed in the right femoral artery, and used to sample arterial blood. The bladder was catheterized via a suprapubic approach using a Foley catheter (14 F), which was connected to a graduated conical flask to measure urine volume. In group A, the aortic flow probe cable was connected to a flowmeter (TS420, Transonic Systems, Ithaca, NY) to obtain CO. A catheter-tipped micro-manometer (SPC-330A, Millar Instruments, Houston, TX) was introduced via the right radial artery into the ascending aorta to measure AP. In group B, a fluid filled catheter (22 gauge) was placed in the right radial artery via a 2-cm skin incision, and connected to a pressure transducer (DX-200, Nihon Kohden, Tokyo, Japan) to measure AP. A pulmonary artery catheter (6F) (T173HF6, Edwards Lifesciences, Irvine, CA) was positioned in the pulmonary artery via the right jugular vein, and used to sample mixed venous blood, and to measure thermo-dilution CO (CO_TD_).

### Echocardiography

Transthoracic echocardiography was performed using an echocardiography system equipped with a transducer operating at frequencies of 3 to 5 MHz (Artida, Toshiba, Tokyo, Japan) [17, 18]. The transducer was directed from the apex to obtain an apical 4-chamber view [17]. The tricuspid and mitral annular velocities were obtained once in each animal using pulsed tissue Doppler technique to determine systolic velocity of the tricuspid annulus (s’_T_) and that of the mitral annulus (s’_M_) as described previously [17]. The s’_T_ and s’_M_ values were used to compute PWP (see Appendix 1, Additional file 1).

In group B, the ascending aortic cross-sectional area was measured from the parasternal short-axis image. Flow velocity of the ascending aorta was acquired using continuous wave Doppler technique (emitting frequency, 3.3 MHz) with the transducer directed from the apex and held using a mechanical arm [18]. Audio output signal encoding Doppler-shifted frequency was output continuously from the echocardiography system [18]. Analog signals of the audio output, AP, and ECG were digitized (20 kHz, 16-bit) by a laboratory computer (Computer 2) (ME-B, NEC, Tokyo, Japan), and analyzed on-line to compute CO with use of the aortic cross-sectional area as reported previously [18]. CO was continuously output from Computer 2 to Computer 1.

### Hemodynamic data acquisition, processing, and command generation

Analog signals of AP, CO, and CVP were digitized (200 Hz,12-bit) by Computer 1, and stored on a hard disk for off-line analysis. The digitized signals of AP, CO, and CVP were smoothed by a low-pass filter with a time constant 10 s, and used as control variables for the system (see Fig. 1a). If the feedback loops were closed, simultaneously with the analogue to digital signal conversion, i.e. every 5 ms, Computer 1 calculated the infusion rate of NA using Ki and Kp, determined the on/off status of the RiA infusion at 15 ml·min^−1^ based on the “if-then” rules, and sent command signals to the pumps.

### Measurements of blood lactate and hemoglobin oxygen saturation

Arterial blood samples were analyzed for lactate concentration using a hand-held analyzer (Lactate Pro 2; Arkray KDK, Kyoto, Japan) [19], and for hematocrit (Ht) and oxygen saturation (SaO_2_) using a blood gas analyzer (IRMA Trupoint Blood Gas Analysis System, Edison, NJ) [20].

In group B, mixed venous blood samples were analyzed for oxygen saturation (SvO_2_).

### Experimental protocols

After all the preparations were completed, 30 min were allowed for stabilization (Baseline data acquired at the end of stabilization). Acute endotoxemia was induced by infusion of *Escherichia coli* lipopolysaccharide (LPS) (055:B5; Sigma Laboratories, St. Louis, MO) at a concentration of 10 mg·ml^−1^ in saline. In each animal, 4 mg·kg^−1^ of LPS was infused through the right femoral vein catheter over 60 min.

After the induction of endotoxemia (Shock data acquired at the end of 60-min endotoxin infusion), we applied the newly developed system to the animals. AP* was defined as 70 mmHg [1]. CO* was defined as 110–140 ml·min^−1^·kg^−1^. AP* and CO* were fed into the system. The system was activated by closing the feedback loops of NA infusion and RiA infusion. The infusion rates of NA and RiA were stored in Computer 1. Urine volume during the closed-loop control was recorded. We supervised the performance of the system until 4 h after activation (Resuscitated data acquired at the end of 4-h operation of the system).

In group B, we measured CO_TD_ by thermodilution using the pulmonary artery catheter at baseline, after the induction of endotoxin shock, and every 1 h during the activation of the system [11].

### Data analysis and statistics

To evaluate the rapidity of the control of AP and CO by the new system, we calculated response time required for AP and CO to reach respective acceptable ranges, which were defined as AP*-5 mmHg, i.e. 65 mmHg, for AP [1] and CO* - 10 ml·min^−1^·kg^−1^ for CO, respectively. To evaluate the precision of the control of AP and CO by the new system, we analyzed performance error [21, 22]. In each animal, the following parameters were calculated for AP and CO: (1) percentage performance error (PE; defined as the difference between each measured value and the target value, expressed as a percentage of the target value); (2) median performance error (MDPE; defined as the median of all values of PE); (3) median absolute performance error (MDAPE; defined as the median of the absolute values of PE [|PE|]); (4) wobble (a measure of the variability of PE around MDPE, calculated as the median value of the differences between each value of PE and MDPE); and (5) divergence (a measure of the trend of change in |PE| with time). Derivation of these parameters has been described previously [21, 22]. Since a steady state was reached within 1 h after activation of the system, PE were determined every minute from 1 to 4 h after activation.

Group data are expressed as median (interquartile range). The level of statistical significance was defined as *P* < 0.05. Mann-Whitney U test was used to compare parameters between different groups. Friedman test followed by the Wilcoxon signed rank test was used to compare variables or parameters among 3 time points (Baseline, Shock, and Resuscitated), where a Bonferroni correction was applied to maintain α at 0.05 such that the significance criterion was *P* < 0.0167 (0.05/3) [23]. We used the non-parametric Spearman correlation coefficient to examine associations between variables, and interpreted the correlations coefficients using Cohen’s conventions [23] (.10 small, .30 moderate, .50 large). To determine 99% confidence interval (CI) of the difference between variables, we used a bootstrap technique (1000 replicates) [17, 24]. Statistical analyses were performed using commercially available software (Statistica, Statsoft, Inc., Tulsa, OK, USA).

## Results

Intravenous LPS induced endotoxin shock in all 8 animals (Baseline vs Shock in Table 1). After induction of endotoxin shock, AP decreased significantly from 95 (91–108) to 43 (39–45) mmHg (99% CI of difference, −81 to −40 mmHg; *P* = 0.012), CO decreased significantly from 112 (104–142) to 62 (51–73) ml·min^−1^·kg^−1^ (99% CI of difference, −96 to −41 ml·min^−1^·kg^−1^; *P* = 0.012), while heart rate increased significantly from 115 (111–140) to 161 (141–176) bpm (99% CI of difference, 17–71 bpm; P = 0.012). As for the cardiovascular parameters, V decreased significantly from 22 (17–38) to 11 (10–22) ml·kg^−1^ (99% CI of difference, −27 to −6 ml·kg^−1^; P = 0.012), S decreased significantly from 52 (38–55) to 27 (18–31) ml·min^−1^·kg^−1^ (99% CI of difference, −36 to −12 ml·min^−1^·kg^−1^; P = 0.012), while R did not change significantly. Blood lactate level increased significantly from 1.8 (1.6–1.9) to 3.0 (2.5–3.5) mmol·L^−1^ (99% CI of difference, 0.6–2.1 mmol·L^−1^; P = 0.012), Ht increased significantly from 30 (29–31) to 43 (42–45) % (99% CI of difference, 11–16%; P = 0.012), while SaO_2_ decreased significantly from 99 (99–100) to 98 (95–98) % (99% CI of difference, −5.2 to −0.7%; P = 0.012). In group B, SvO_2_ did not change significantly.

**Table 1:** (Comparison of variables and parameters at different time points)

	Baseline	Shock	Resuscitated	*P* Value		
				Baseline versus Shock	Shock versus Resuscitated	Baseline versus Resuscitated
Heart Rate (bpm)	115 (111–140)	161 (141–176)	149 (133–177)	0.012	0.263	0.050
AP (mmHg)	95 (91–108)	43 (39–45)	70 (69–71)	0.012	0.012	0.012
CO (ml·min^−1^·kg^−1^)	112 (104–142)	62 (51–73)	130 (125–138)	0.012	0.012	0.401
CVP (mmHg)	2 (0–6)	1 (0–4)	6 (3–7)	0.050	0.012	0.017
PWP (mmHg)	8 (5–11)	6 (5–9)	11 (8–4)	0.036	0.012	0.017
R (mmHg·min·kg·ml^−1^)	0.88 (0.69–1.00)	0.74 (0.55–0.78)	0.51 (0.46–0.51)	0.263	0.012	0.017
V (ml·kg^−1^)	22 (17–38)	11 (10–22)	37 (29–39)	0.012	0.012	0.025
S (ml·min^−1^·kg^−1^)	52 (38–55)	27 (18–31)	43 (39–48)	0.012	0.012	0.208
Blood lactate (mmol·L^−1^)	1.8 (1.6–1.9)	3.0 (2.5–3.5)	3.2 (2.4–4.3)	0.012	0.735	0.012
Ht (%)	30 (29–31)	43 (42–45)	38 (35–45)	0.012	0.263	0.069
SaO2 (%)	99 (99–100)	98 (95–98)	98 (97–99)	0.012	0.401	0.012
SvO2 (%)	85 (83–87)	72 (67–76)	82 (77–86)	0.068	0.068	1.000

Values are median (interquartile range). *AP* mean arterial pressure, *CO* cardiac output, *CVP* central venous pressure, *PWP* pulmonary wedge pressure, *R* systemic arterial resistance, *V* stressed blood volume, *S* Frank-Starling slope of the left ventricle, *Ht* hematocrit, *SaO*_*2*_ arterial oxygen saturation, *SvO*_*2*_ mixed venous oxygen saturation. (n = 8 for each time point, except for SvO_2_ with n = 4 for each time point)

**Fig. 2:**
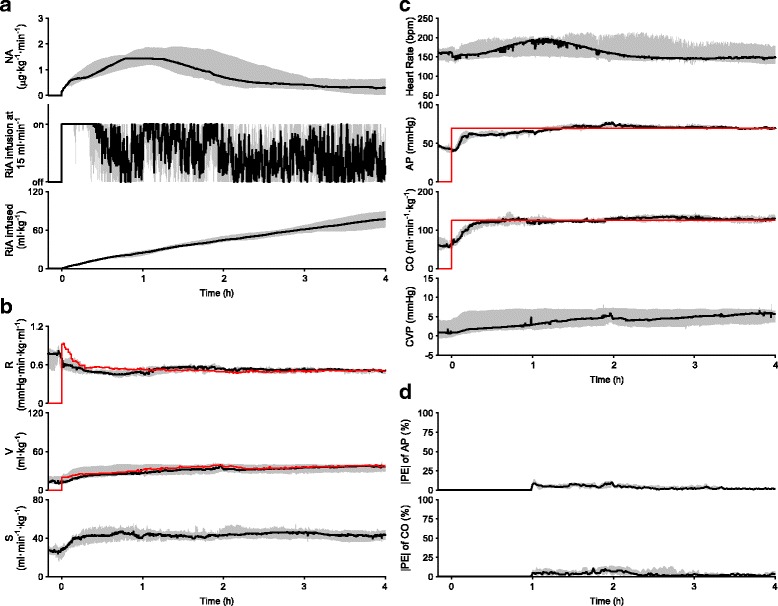
Closed-loop control of hemodynamic resuscitation in 8 dogs over the period of 4 h. Data are expressed as median (solid line) and interquartile range (gray area). **a** Time courses of NA infusion rate, on/off status of RiA infusion at 15 ml·min^−1^, and cumulated volume of RiA infused. **b** Time courses of cardiovascular parameters. Red lines indicate median target parameters (top; R*, middle; V*). R* and V* were recalculated every minute. **c** Time courses of hemodynamic variables. Red lines indicate median target hemodynamic variables (second from the top; AP*, third from the top; CO*). AP* and CO* were constant throughout the control period. **d** Time courses of absolute performance error (|PE|) for AP and CO

Figure 2 summarizes the results of hemodynamic resuscitation obtained from 8 dogs. The automated system was activated at 0 h. Fig. 2a shows the time course of the NA infusion rate, the on/off status of the RiA infusion at 15 ml·min^−1^, and the cumulated volume of RiA infused. In all the animals, NA and RiA infusion were started at the time of system activation (Fig. 2a). Time-averaged infusion rate of NA over the period of 4 h was 0.8 (0.5–1.1) μg·kg^−1^·min^−1^. In two animals, infusion rate of NA was temporarily increased more than 0.5 μg·kg^−1^·min^−1^, but was reduced to zero by the end of the period of 4 h. Total volume of RiA infused was 77 (64–89) ml·kg^−1^. Fig. 2b shows the time courses of R, V, and S. Once the system was activated, R initially decreased in all the animals to a variable degree. R and V gradually approached their respective target values. In all the animals, AP and CO were controlled at their respective target levels (Fig. 2c). Fig. 2d shows the time courses of |PE| in AP and CO. |PE| in AP and CO decreased to less than 5% within 3 h after activation of the system. As summarized in Table 2, response times of AP and CO in the 8 animals were 29 (10–51) min and 16 (14–18) min, respectively. MDAPE in AP and CO were 2.5 (2.1–4.5) and 2.4 (1.4–5.5) %, respectively. At 4 h of hemodynamic resuscitation, AP and CO were significantly increased to 70 (69–71) mmHg (99% CI of difference, 22–36 mmHg; *P* = 0.012) and 130 (125–138) ml·min^−1^·kg^−1^ (99% CI of difference, 54–97 ml·min^−1^·kg^−1^; P = 0.012), respectively, but blood lactate level, Ht and SaO_2_ were not significantly different from those observed before resuscitation (Shock vs Resuscitated in Table 1). The urine output was 5 (2–6) ml·kg^−1^ over the period of 4 h.

**Table 2:** (Rapidity and precision of control of AP and CO)

	Response time	MDPE	MDAPE	Wobble	Divergence
	(min)	(%)	(%)	(%)	(%·min^−1^)
AP					
Total animals (*n* = 8)	29 (10–51)	1.3 (0.5–2.1)	2.5 (2.1–4.5)	2.9 (2.0–4.1)	−0.03 (−0.04 to −0.02)
Group A (n = 4)	30 (10–51)	2.0 (1.3–3.3)	4.4 (2.2–7.4)	4.2 (2.6–6.1)	−0.03 (−0.05 to −0.02)
Group B (*n* = 4)	29 (19–46)	0.7 (0.0–1.3)	2.4 (2.1–2.9)	2.4 (2.0–3.0)	−0.03 (−0.04 to −0.02)
*P* Value (Group A versus B)	0.886	0.200	0.686	0.486	1.000
CO					
Total animals (n = 8)	16 (14–18)	0.7 (0.2–3.4)	2.4 (1.4–5.5)	1.7 (1.2–5.0)	−0.01 (−0.03 to −0.01)
Group A (n = 4)	18 (15–47)	1.7 (0.1–5.7)	2.1 (1.2–5.8)	1.7 (1.1–3.2)	−0.02 (−0.05 to 0.01)
Group B (n = 4)	15 (14–15)	0.7 (0.2–2.0)	3.6 (1.7–5.5)	3.1 (1.3–5.0)	−0.01 (−0.02 to −0.01)
*P* Value (Group A versus B)	0.343	1.000	0.886	0.886	1.000

Values are median (interquartile range), *AP* mean arterial pressure, *CO* cardiac output, *MDPE* median performance error, *MDAPE* median absolute performance error

**Fig. 3:**
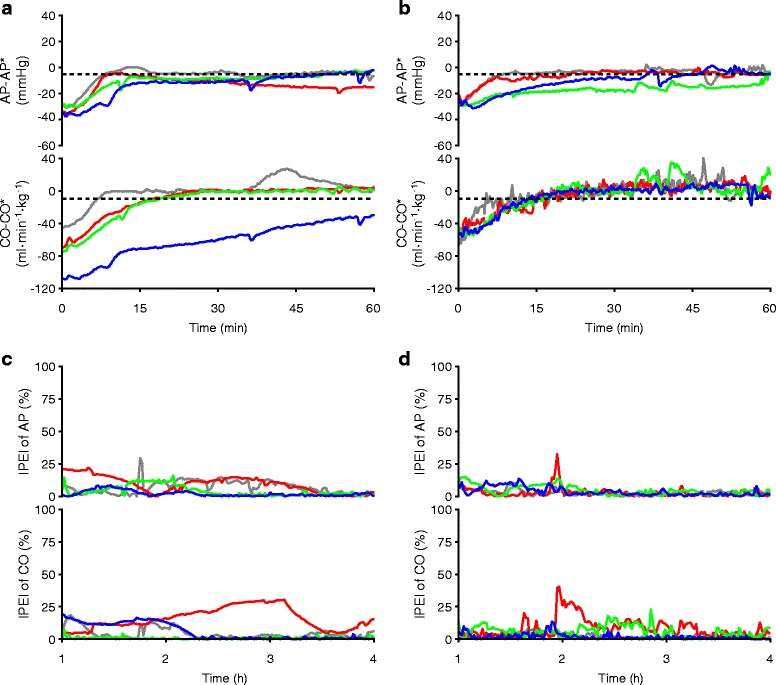
Effectiveness of control of AP and CO in each animal in group A and B. Data of each dog is color-coded as shown in the panels. **a** Time courses of AP-AP*, and CO-CO* in group A. **b** Time courses of AP-AP*, and CO-CO* in group B. Broken lines in **a** and **b** correspond to predefined acceptable ranges. **c** Time courses of |PE| for AP and CO in group A. **d** Time courses of |PE| for AP and CO in group B

There were no significant differences between group A and B in time-averaged infusion rate of NA over the period of 4 h [group A: 0.8 (0.6–1.2) μg·kg^−1^·min^−1^ versus group B: 0.7 (0.5–1.1) μg·kg^−1^·min^−1^], and in total volume of RiA infused [group A: 71 (58–88) ml·kg^−1^ versus group B: 84 (74–89) ml·kg^−1^]. To highlight the rapidity of control of AP and CO, time courses of AP-AP*, and those of CO-CO* during 1st h of closed-loop control are shown for each animal in group A (Fig. 3a) and B (Fig. 3b), where response times were the durations for AP and CO to reach the horizontal broken lines. Response times were less than 60 min, except in two animals, in CO control in group A (blue line in Fig. 3a, 75 min) and in AP control in group B (green line in Fig. 3b, 131 min). There were no statistically significant difference in response times of AP and CO between group A and B (Table 2). To highlight the precision of control of AP and CO, time courses of |PE| in AP and CO from 1 to 4 h after activation of the system are shown for each animal in group A (Fig. 3c) and B (Fig. 3d). There were no statistically significant difference in any of the PE parameters for AP and CO between group A and B (Table 2).

**Fig. 4:**
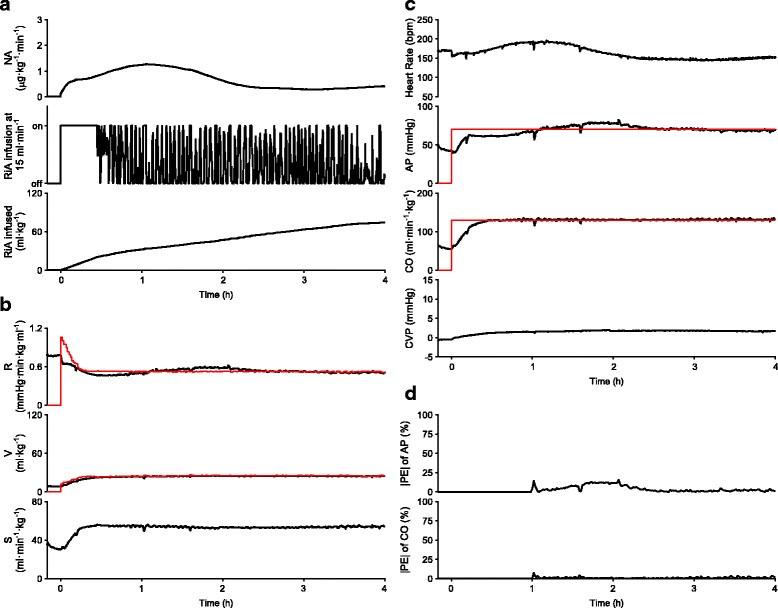
Closed-loop control of hemodynamic resuscitation in one dog in group A over the period of 4 h. **a** Time courses of NA infusion rate, on/off status of RiA infusion at 15 ml·min^−1^, and cumulated volume of RiA infused. **b** Time courses of cardiovascular parameters. Red lines indicate target parameters (top; R*, middle; V*). R* and V* were recalculated every minute. **c** Time courses of hemodynamic variables. Red lines indicate target hemodynamic variables (second from the top; AP*, third from the top; CO*). AP* and CO* were constant throughout the control period. **d** Time courses of |PE| for AP and CO

Figure 4 shows the experimental data of one animal in group A. Early after the activation of the system, R initially decreased (Fig. 4b). Infusion of NA (Fig. 4a) was adjusted so that R approached to R*. Until about 30 min after activation of the system, RiA infusion was continuously activated, since V was lower than V* (Fig. 4a, b). Once V was controlled to V*, the status of RiA infusion was changed between “on” and “off” so that V was maintained at V*. Data of the cumulated volume of RiA infused at 4 h indicates total volume of RiA infused in this animal (75 ml·kg^−1^). Concomitantly with these changes in R and V, S improved. By controlling the cardiovascular parameters, the automated system controlled AP and CO as demonstrated in Fig. 4c. Response times of AP and CO were 51 and 19 min, respectively. MDAPE in AP and CO were 2.5 and 0.8%, respectively (Fig. 4d).

**Fig. 5:**
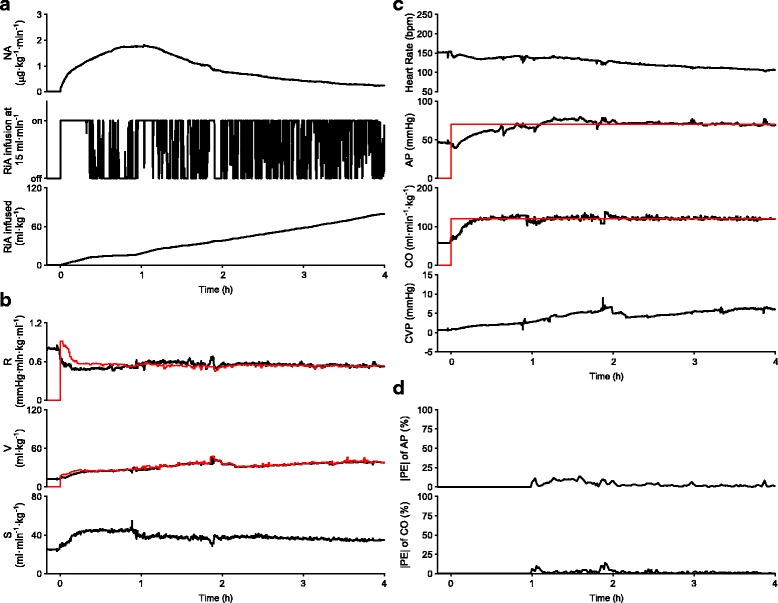
Closed-loop control of hemodynamic resuscitation in one dog in group B over the period of 4 h. **a** Time courses of NA infusion rate, on/off status of RiA infusion at 15 ml·min^−1^, and cumulated volume of RiA infused. **b** Time courses of cardiovascular parameters. Red lines indicate target parameters (top; R*, middle; V*). R* and V* were recalculated every minute. **c** Time courses of hemodynamic variables. Red lines indicate target hemodynamic variables (second from the top; AP*, third from the top; CO*). AP* and CO* were constant throughout the control period. **d** Time courses of |PE| for AP and CO

Figure 5 shows the experimental data in one animal in group B. Overall, the time courses of the NA infusion rate, the on/off status of the RiA infusion at 15 ml·min^−1^, and the cumulated volume of RiA infused (Fig. 5a), and the time courses of R, V and S (Fig. 5b) were similar to those seen in the animal in Fig. 4 (group A). Total volume of RiA infused was 80 ml·kg^−1^. By controlling the cardiovascular parameters, the automated system restored AP and CO to their respective target levels. Response times of AP and CO were 37 and 13 min, respectively (Fig. 5c). MDAPE in AP and CO were 2.2 and 1.3%, respectively (Fig. 5d).

**Fig. 6:**
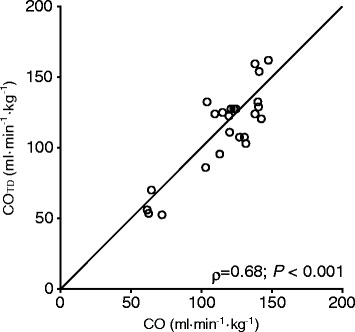
Association between CO measured less invasively and thermo-dilution CO (CO_TD_) in group B. Line of identity, Spearman Rank-Order Correlation Coefficient (ρ), and probability value are shown

In Group B, CO measured less invasively by our system and CO_TD_ were significantly correlated (*P* < 0.001) with large Spearman correlation efficient (*ρ* = 0.68) (Fig. 6).

## Discussion

To the best of our knowledge, we are the first to succeed in automated closed-loop control of hemodynamic resuscitation in endotoxin shock. Rapidity of control of AP by this system satisfies the SSC guidelines, which recommend that AP should be recovered to more than 65 mmHg within the initial 6 h of hemodynamic resuscitation [1]. Rapidity of control of CO by this system seems acceptable in that CO was restored to baseline level within 4 h after activation of the system. Precision of control of AP and CO were evaluated by the PE parameters (Table 2). MDAPE in AP and CO were 2.5 and 2.4%, respectively, which are smaller than that reported previously in closed-loop hemodynamic control by other groups [22, 25]. Other PE parameters of AP and CO were comparable to or smaller than those reported previously [22, 25]. Furthermore, even when the system was modified to a less invasive, clinically feasible version, rapidity and precision of control of AP and CO were not worsened. In this study, although we observed performance of this system during 4 h period, the period may be extended without difficulty in clinical settings until the infection causing sepsis is resolved. This system may be a powerful clinical tool in rescuing patients with septic shock.

In the present study, we extended the system that we developed for the control of AP, CO and PWP in heart failure to control AP and CO in septic shock. Since the number of variables to be controlled was reduced from 3 to 2, the present success might be predictable. However, the hemodynamic pathophysiology is distinctly different between heart failure and septic shock. Generally, heart failure is characterized by increased R and V [26], while septic shock by decreased R and V [1, 8, 15]. Furthermore, the types of drugs used and the responses of R and V to the drugs are distinctly different between these two conditions. These are the major reasons for conducting this preclinical study before the system can be considered for clinical application in patients with septic shock.

This system directly controls R and V, thereby achieving target values for AP and CO. In other words, this system controls the mechanical determinants of AP and CO. This is the unique characteristics of this system compared to the closed-loop drug infusion systems developed by other groups. All other systems attempted to control AP [8, 22] or CO only [27] on the basis of apparent responses of the hemodynamic variables to the drugs. These approaches may work when controlling a single variable with use of a single drug, i.e. AP or CO alone with use of NA or fluid. Comparing to these approaches, our approach is more efficacious in controlling multiple hemodynamic variables using multiple drugs, such as controlling AP and CO using NA and RiA. Since these agents affects AP and CO simultaneously and interactively, such interactions make it difficult to control AP and CO independently based on the apparent drug responses [9].

Following the induction of endotoxin shock, V and S decreased significantly, while R decreased moderately, but not significantly (Table 1). Once activated, this system initiated RiA infusion to increase V to the target value and maintain the target level. Total volume of RiA infused was much larger than the net increase in V (Fig. 2a vs 2b). This phenomenon is firstly due to the low plasma expanding potency of crystalloid solutions such as RiA [28]. Second, endothelial damage induced by endotoxin may increase plasma leakage [29], and consequently require continuous compensation from RiA infusion to maintain V at its target value. This speculation is not contradictory to the observation that Ht, an indirect marker of plasma leakage [29], increased after endotoxin injection, and did not recover to baseline level even after fluid supplementation. The persistent elevation of Ht might be attributable to other mechanisms such as enhanced recruitment of red blood cells from spleen stimulated by endotoxin, or by infused NA [30, 31]. The mechanisms of the persistent elevation of Ht remain to be unveiled. In any way, infusion of large amount of fluids was associated with poor prognosis in sepsis patients [32]. If patients require substantial amount of fluids, infusion of albumin solution, as an alternative to RiA infusion, may be preferred in our system [1].

Early after system activation, R initially decreased in all the animals (Figs. 2b, 4b, and 5b), when infusion rate of NA was minimum and being gradually increased (Figs. 2a, 4a, and 5a). This early reduction in R was most likely induced by hemodilution accompanying RiA infusion [33]. Thereafter, R recovered gradually and was controlled at the target value by NA infusion. Although S was not selected as a control parameter, S also increased after system activation (Figs. 2b, 4b, and 5b). S is related to R, left ventricular end-systolic elastance (E_es_, an index of LV contractility), heart rate (HR) and diastolic myocardial stiffness (κ) by the following formula [10, 11, 13],

$$ \mathrm{S}={\mathrm{E}}_{\mathrm{es}}/\upkappa /\left({\mathrm{E}}_{\mathrm{es}}/\mathrm{HR}+\mathrm{R}\right) $$

This formula suggests that increase in S observed after system activation was probably due to enhanced cardiac contractility, E_es_, through beta-adrenergic stimulation by NA [16] and reduced R accompanying RiA infusion [33]. This increase in S, an upward shift in Flank-Starling curve, more or less contributed to rapid restoration of AP and CO. Since relative gain in S was apparently larger than that in R (Fig. 2b), it would seem more efficacious to select S, and not R, as the parameter to be controlled by NA infusion, However, according to the framework of circulatory equilibrium [9, 12, 13], if S and V, but not R, are used as control parameters, it would be possible to control CO, but impossible to independently control AP.

In this study, we did not compare the efficacy of closed-loop control of hemodynamic resuscitation by our system with that of manual control by care providers as was done previously [27]. The reason is that this system is not intended to replace care providers, but is intended to be used under supervision by care providers. However, in future, comparison of the closed-loop hemodynamic control by our system with the manual control by the providers will be required to make inferences on how our approach compares to clinically established practice.

In group B, CO measured less invasively showed significant correlation with CO_TD_. Our previous study [18] indicated that an initial calibration with some reference method is desired for absolute accuracy of the less invasive CO measurement. However, routine use of the pulmonary artery catheter is not recommended in patients with sepsis [1]. Precise presetting including measurements of aortic cross-sectional area, and aligning the ultrasound Doppler beam along the aortic flow may be mandatory when our less invasive CO measurements are applied to patients with difficulty in the initial calibration with reference CO.

### Clinical perspective

Our system may be used for early hemodynamic resuscitation as well as for weaning from hemodynamic support. No closed-loop control systems for early resuscitation of septic shock have been reported. Only one clinical trial reported that closed-loop control for weaning from NA infusion in septic patients has beneficial effects on clinical outcomes [8]. We did not systematically evaluate the weaning of drug infusion in this study, since it would require observation period far longer than 4 h. In the clinical trial [8], the weaning from NA infusion took more than 24 h. However, in principle, the negative feedback mechanisms used in our system automatically quit drug infusions once they are no longer required. Indeed, we observed that NA infusion was quitted by the end of 4 h period in 2 out of the 8 animals. We believe that closed-loop control of hemodynamic resuscitation by our system will further enhance the improvement in outcomes demonstrated in that clinical trial. From the viewpoint of clinical care, our system will reduce the stress and work imposed on the care providers who are managing patients with septic shock. The care providers will be able to spend more time on other patient-related activities, thereby improving the quality of patient care [7].

Optimization of macro-circulatory endpoints including AP, CO and global oxygen delivery is an initial step in the hemodynamic resuscitation of patients with septic shock [1]. Our system automates this initial step. The next step of resuscitation is to assess the adequacy of organ perfusion indicated by micro-circulatory resuscitation targets such as optimization of blood lactate concentration. However, optimization of macro-circulation does not necessary guarantee optimization of micro-circulation [34]. Indeed, in animals resuscitated by our system, blood lactate was not normalized despite the achievement of optimization of macro-circulatory endpoints. SSC guidelines recommend that blood lactate level should be normalized in initial resuscitation [1]. However, recent clinical trials in patients with septic shock noted that blood lactate level is not normalized until 24–48 h post resuscitation [2, 3]. In the dogs in this study, duration of the closed-loop control longer than 4 h might be needed to confirm restoration of basal lactate level.

### Limitation

We estimated PWP with use of the previously developed technique [17], which uses CVP and the ratio of the tissue-Doppler tricuspid to mitral annular velocities. However, the accuracy of this technique has not yet been confirmed in subjects with endotoxemia. Endotoxin has been shown to change the mechanical properties of the pulmonary artery and vein [35]. This can adversely affect the reliability of our PWP estimation technique. Further studies on these respects are required in future.

Endotoxin administration is commonly used in animal models of sepsis, since endotoxin under some circumstances plays an important role in the pathogenesis of sepsis [36, 37]. However, there are several concerns that the infusion of endotoxin is not a suitable model with which to simulate sepsis/septic shock. Time course of canine endotoxin shock is generally different from human sepsis, with animals more often showing rapid onset of circulatory collapse [36, 37]. Although gram-positive bacteria are detected as causative organisms as frequently as gram-negative ones in patients with septic shock [2, 3, 8], endotoxin is released only by gram-negative bacteria, but not by gram-positive ones. The use of corticosteroids and anti-TNF-α has been effective in animal models of endotoxemia, but has failed in clinical trials [36, 37].

Infusion of RiA reduced R early after system activation. This can be an adverse interaction between the two feedback loops (Fig. 1a), where the input of one loop (RiA) can reduce feedback gain in another loop (R in response to NA). This may cause system malfunction, where NA infusion rate may be increased infinitely. Fortunately, in this study, we did not observe such malfunction. However, this may become problem when our system is applied to subjects showing R with extremely low sensitivity to NA.

Severe myocardial depression occurs in 20 to 50% of patients with septic shock, and is characterized by blunted ability to enhance cardiac contractility despite increased levels of catecholamine [38]. This may adversely affect the hemodynamic resuscitation by this system, and require a closed-loop controller of S with inotrope, in addition to controllers of R and V. Indeed, about 5% of patients with septic shock required inotropes in addition to fluids and vasopressors to restore hemodynamics [2, 3].

Furthermore, we applied the hemodynamic support 1 h after the onset of symptoms, although such early intervention is desirable but rarely achieved in clinical settings.

A specific limitation we had is that we did not perform a splenectomy, and/or evaluations of plasma volume/total blood volume using the dye-dilution methods, which would have better unveiled the mechanisms of the persistent increase in Ht [30, 31].

## Conclusion

We have developed a closed-loop drug infusion system for automated hemodynamic resuscitation in septic shock. In a canine model of endotoxin shock, our system automatically restored and precisely maintained AP and CO at their target values with small performance error. Our system is potentially an attractive clinical tool for rescuing patients with septic shock.

## Additional files

Additional file 1: Calculation of hemodynamic variables and parameters. (DOCX 29 kb)

Additional file 2: Calculation of target hemodynamic variables and parameters. (DOCX 28 kb)

Additional file 3: Response of R to NA infusion. (DOCX 43 kb)

## Abbreviations

AP: Arterial pressure
AP*: target value for AP
CO: Cardiac output
CO*: target value for CO
CO**: Effective CO*
CO_TD_: thermo-dilution CO
CVP: Central venous pressure
CVP*: target value for CVP
Ki: integral gain
Kp: proportional gain
MDAPE: Median absolute performance error
MDPE: Median performance error
NA: Noradrenaline
PE: Percentage performance error
|PE|: absolute value of PE
PWP: Pulmonary wedge pressure
PWP*: target value for PWP
R: systemic arterial resistance
R*: target value for R
RiA: Ringer’s acetate
S: Frank-Starling slope of the left ventricle
s’_M_: Systolic velocity of the mitral annulus
s’_T_: systolic velocity of the tricuspid annulus
SSC: Surviving Sepsis Campaign
V: total stressed blood volume
V*: target value for V
δR: change in R from baseline
ΔR: difference between R* and R
ΔV: difference between V* and V
