# Supplementary material for: Computer-controlled closed-loop drug infusion system for automated hemodynamic resuscitation in endotoxin-induced shock
Source: BMC Anesthesiol. 2017 Oct 23;17:145. doi: 10.1186/s12871-017-0437-9 (PMC5654105; doi:10.1186/s12871-017-0437-9)
Supplement: Supplementary file 1 — Calculation of hemodynamic variables and parameters. (DOCX 29 kb) [file 12871_2017_437_MOESM1_ESM.docx]

**Appendix 1. Calculation of hemodynamic variables and parameters**

In the system (Fig. 1a in the main manuscript), PWP is estimated from CVP, systolic velocity of the tricuspid annulus (s’_T_) and that of the mitral annulus (s’_M_) as follows [1],

PWP=1.1×CVP×s’_T_/s’_M_+5.3 (A1)

CVP is continuously acquired in monitoring of PWP, while s’_T_, and s’_M_ are determined once in each subject using echocardiography [1].

Based on the circulatory equilibrium framework [2,3,4], the system calculates subject’s R, V and S (blue rectangle in Fig. 1a in the main manuscript).

R is calculated from AP, CO, and CVP by the following formula:

R = (AP- CVP)/CO (A2)

V is calculated from CO, CVP and PWP as follows [3]:

V = (CO + 19.61×CVP + 3.49×PWP) ×0.129 (A3)

S is calculated from CO and PWP as follows [2,4]:

S = CO/[ln(PWP -2.03)+0.80] (A4)

Reference in Appendix 1

1. Uemura K, Inagaki M, Zheng C, Li M, Kawada T, Sugimachi M. A novel technique to predict pulmonary capillary wedge pressure utilizing central venous pressure and tissue Doppler tricuspid/mitral annular velocities. Heart Vessels. 2015;30:516-26.
2. Uemura K, Kamiya A, Hidaka I, Kawada T, Shimizu S, Shishido T, Yoshizawa M, Sugimachi M, Sunagawa K. Automated drug delivery system to control systemic arterial pressure, cardiac output, and left heart filling pressure in acute decompensated heart failure. J Appl Physiol. 2006;100:1278-86.
3. Uemura K, Sugimachi M, Kawada T, Kamiya A, Jin Y, Kashihara K, Sunagawa K. A novel framework of circulatory equilibrium. Am J Physiol Heart Circ Physiol. 2004;286:H2376-85.
4. Uemura K, Kawada T, Kamiya A, Aiba T, Hidaka I, Sunagawa K, and Sugimachi M. Prediction of circulatory equilibrium in response to changes in stressed blood volume. Am J Physiol Heart Circ Physiol. 2005;289:H301-7.
