# Supplementary material for: Computer-controlled closed-loop drug infusion system for automated hemodynamic resuscitation in endotoxin-induced shock
Source: BMC Anesthesiol. 2017 Oct 23;17:145. doi: 10.1186/s12871-017-0437-9 (PMC5654105; doi:10.1186/s12871-017-0437-9)
Supplement: Supplementary file 2 — Calculation of target hemodynamic variables and parameters. (DOCX 28 kb) [file 12871_2017_437_MOESM2_ESM.docx]

**Appendix 2. Calculation of target hemodynamic variables and parameters**

Based on AP*, CO*, and subject’s CO and S, the system calculates R* and V* (red rectangle in Fig. 1a in the main manuscript). The system first calculates effective CO* (CO**) by the following “if-then” rules:

If CO + 10 ml·min^-1^·kg^-1^ < CO* then CO** = CO + 10 ml·min^-1^·kg^-1^. (A5)

If CO + 10 ml·min^-1^·kg^-1^ ≥ CO* ≥ CO then CO** = CO*. (A6)

If CO > CO* then CO** = CO. (A7)

The system calculates the target values for PWP (PWP*) from CO** and S by back calculation using Eq. (A4) in Appendix 1, as well as the target value for CVP (CVP*) from PWP* by back calculation using Eq. (A1) in Appendix 1. The system calculates R* from AP*, CO**, and CVP* using Eq. (A2) in Appendix 1, and V* from CO**, CVP*, and PWP* using Eq. (A3) in Appendix 1.
