# Supplementary material for: Computer-controlled closed-loop drug infusion system for automated hemodynamic resuscitation in endotoxin-induced shock
Source: BMC Anesthesiol. 2017 Oct 23;17:145. doi: 10.1186/s12871-017-0437-9 (PMC5654105; doi:10.1186/s12871-017-0437-9)
Supplement: Supplementary file 3 — Response of R to NA infusion. (DOCX 43 kb) [file 12871_2017_437_MOESM3_ESM.docx]

**Appendix 3. Response of R to NA infusion**

In preliminary experiments, we examined the response of R to NA infusions in 4 dogs with endotoxin shock. Surgical preparation including implantation of the aortic flow probe using aseptic technique, and measurements of AP, CO and CVP were performed as described in the text. Under open-loop condition, we infused NA at 0.3 μg·kg^-1^·min^-1^ for 10 min to obtain a step response of R [1]. We described the step response by a transfer function of a first-order model with a transport delay. In this model, change in R from baseline (δR) in response to NA infusion can be expressed by the following formula:

$\delta R(t) =\left\{\begin{matrix} G\cdot\left[1-\exp\left(-\frac{t-L}{T}\right)\right] \left(t\geq L\right) \\ 0 \left(t<L\right) \end{matrix}\right.$ (A8)

where G is static gain [mmHg·min·kg·ml^-1^ (μg·kg^-1^·min^-1^)^-1^], L is transport delay (sec), and T is time constant (sec). We estimated the parameters G, L, and T by approximating δR to Eq. A8 using the least square method [1]. Fig. A1 shows the time course of δR during NA infusion (n=4). NA infusion increased δR exponentially. Averaged values of G, L, and T were 0.33 mmHg·min·kg·ml^-1^ (μg·kg^-1^·min^-1^)^-1^, 74 sec, and 62 sec, respectively. Using these parameters [1], we defined gain constants, Ki = 0.83 sec^-1^ and Kp = 0.42 μg·kg^-1^·min^-1^ (mmHg·min·kg·ml^-1^)^-1^, for the proportional-integral feedback controller of NA infusion (Fig. 1b in the main manuscript).

**Fig. A1** Step response of R to NA infusion (0.3 μg·kg^-1^·min^-1^) (n=4). δR, change in R from baseline. Data are expressed as median (solid line) and interquartile range (gray area).

Reference in Appendix 3.

1. Uemura K, Kamiya A, Hidaka I, Kawada T, Shimizu S, Shishido T, Yoshizawa M, Sugimachi M, Sunagawa K. Automated drug delivery system to control systemic arterial pressure, cardiac output, and left heart filling pressure in acute decompensated heart failure. J Appl Physiol. 2006;100:1278-86.
